# Work of community health agents in the Family Health Strategy: meta-synthesis

**DOI:** 10.11606/S1518-8787.2018052000395

**Published:** 2018-02-07

**Authors:** Carolina Maria do Carmo Alonso, Pascal Daniel Béguin, Francisco José de Castro Moura Duarte

**Affiliations:** I Universidade Federal do Rio de Janeiro. Faculdade de Medicina. Departamento de Terapia Ocupacional. Rio de Janeiro, RJ, Brasil; II Université Lumière Lyon 2. Institut D’Etudes du Travail. UMR 5600-LabEX IMU. Lyon, France; III Universidade Federal do Rio de Janeiro. COPPE. Programa de Engenharia de Produção. Rio de Janeiro, RJ, Brasil

**Keywords:** Community Health Workers, Family Health Strategy, Working Conditions, Health Human Resource Evaluation, Review, Agentes Comunitários de Saúde, Estratégia Saúde da Família, Condições de Trabalho, Avaliação de Recursos Humanos em Saúde, Revisão

## Abstract

**OBJECTIVE:**

To systematize and analyze the evidence from qualitative studies that address the perception of Brazilian Community Health Agents about their work.

**METHODS:**

This is a systematic review of the meta-synthesis type on the work of community health agents, carried out from the Virtual Health Library using the descriptors “*Agente Comunitário de Saúde*” and “*Trabalho*”, in Portuguese. The strategy was constructed by crossing descriptors, using the Boolean operator “AND”, and filtering Brazilian articles, published from 2004 to 2014, which resulted in 129 identified articles. We removed quantitative or quanti-qualitative research articles, essays, debates, literature reviews, reports of experiences, and research that did not include Brazilian Community Health Agents as subjects. Using these criteria, we selected and analyzed 33 studies that allowed us to identify common subjects and differences between them, to group the main conclusions, to classify subjects, and to interpret the content.

**RESULTS:**

The analysis resulted in three thematic units: characteristics of the work of community health agents, problems related to the work of community health agents, and positive aspects of the work of community health agents. On the characteristics, we could see that the work of the community health agents is permeated by the political and social dimensions of the health work with predominant use of light technologies. The main input is the knowledge that this professional obtains with the contact with families, which is developed with home visits. On the problems in the work of community health agents, we could identify the lack of limits in their attributions, poor conditions, obstacles in the relationship with the community and teams, weak professional training, and bureaucracy. The positive aspects we identified were the recognition of the work by families, resolution, bonding, work with peers, and work close to home.

**CONCLUSIONS:**

This review provided an overview of the difficulties and positive aspects that are present in the daily work of community health agents. Given this, we have raised two challenges. The first one refers to how public policy makers need to appropriation the research results and the second one refers to the need to invest in studies that are designed to generate solutions for the difficulties faced by community health agents in their work.

## RESUMO

**OBJETIVO:**

Sistematizar e analisar evidências levantadas por estudos de natureza qualitativa que abordam a percepção do ACS sobre seu trabalho.

**MÉTODOS:**

Revisão sistemática, tipo metassíntese, sobre o trabalho dos agentes comunitários de saúde, realizada a partir da Biblioteca Virtual em Saúde utilizando os descritores “Agente Comunitário de Saúde” e “Trabalho”. A estratégia foi construída cruzando descritores, usando o operador booleano “AND”, e filtrando artigos brasileiros, publicados de 2004 a 2014, resultando em 129 artigos identificados. Foram excluídos artigos de pesquisas quantitativas ou quanti-qualitativas, ensaios, debates, revisões da literatura, relatos de experiências e pesquisas que não incluíram os ACS como sujeitos. Aplicando esses critérios, foram selecionados e analisados 33 estudos que possibilitaram: identificação de temas comuns e diferenças entre eles; agrupamento de principais conclusões; classificação de temas e interpretação de conteúdo.

**RESULTADOS:**

A análise resultou em três unidades temáticas: Características do trabalho dos agentes comunitários de saúde; Problemas relacionados ao trabalho dos agentes comunitários de saúde; Aspectos positivos do trabalho dos agentes comunitários de saúde. Sobre as características, evidenciou-se que o trabalho dos agentes comunitários de saúde é permeado pelas dimensões política e social do trabalho em saúde com uso predominante de tecnologias leves, tendo como principal insumo o conhecimento que esse profissional obtém junto às famílias, sendo a visita domiciliar o palco para o desenvolvimento desse contato. Sobre os problemas no trabalho dos agentes comunitários de saúde, foram identificados: falta de limites em suas atribuições; condições precárias; obstáculos na relação com a comunidade e equipes; fragilidade na formação profissional e burocratização. Os aspectos positivos identificados foram: reconhecimento do trabalho pelas famílias e resolutividade, formação de vínculo, trabalho junto aos pares e perto da residência.

**CONCLUSÕES:**

Essa revisão teceu um panorama sobre as dificuldades e aspectos positivos que se apresentam no cotidiano de trabalho dos agentes comunitários de saúde. Frente a isso, levantou dois desafios. O primeiro se refere à necessidade de apropriação dos resultados das pesquisas pelos formuladores de políticas públicas e, o segundo, à necessidade de investimento em estudos que se voltem para engendrar soluções para as dificuldades enfrentadas pelos agentes comunitários de saúde no seu trabalho.

## INTRODUCTION

According to Tomaz^38^, the responsibilities of Brazilian Community Health Agents (CHA) can be summarized as the identification of risk situations, guidance for families and community, and referral of identified risk cases and situations to other members of health teams.

This means that the work of CHA is to assist in the planning and implementation of health actions both locally, by forwarding information from the health territory to the Family Health Strategy (FHS), and nationally, by feeding data to information systems of the Ministry of Health^29^.

This shows that CHA play an important role in the expansion and consolidation of primary health care (PHC) and are the subject of studies, among which we highlight, in this article, those focused on the investigation of how they perceive their work.

The relevance of a study on the perspective of workers is related to how services are conceived in a sphere in which players are thought abstractly, with generic characteristics, elaborated from theoretical conceptions. Until the service starts, workers and users will have few opportunities to position themselves on the rules and to try the process to verify difficulties or problems in the operation^8^ ^,^ ^16^ ^,^ ^34^.

In the case of public health services, such as the FHS, this situation is intensified by the distance between the front line and the regulatory agency. This distance hinders the identification of problems that occur in the operation of the service by those who could solve them, while at the same time workers are unaware of what managers expect from the actions at the front line. Such condition results in a mutual incomprehension that, in turn, reduces the chances of correcting eventual gaps in the service project^8^ ^,^ ^34^.

Thus, research studies that have explored the perception of front-line workers, such as CHA, expose the problems of work inadequacy caused by production system projects, processes, work organization, and tasks made from simplifying stereotypes^27^.

In this perspective, this article aims to systematize and analyze evidence from qualitative studies, carried out from 2004 to 2014, which discuss the perception of CHA about their work, offering evidence that can support the improvement of their work process.

## METHODS

This article is a meta-synthesis review on studies on Brazilian CHA linked to the FHS, published between 2004 and 2014, which were selected from the Virtual Health Library (BIREME) that brings together Health Sciences databases, such as the Latin American and Caribbean Literature in Health Sciences (LILACS), the Scientific Electronic Library Online (SciELO), and the Medical Literature Analysis and Retrieve System Online (Medline). We chose this model because the meta-synthesis is a methodological approach used for the rigorous study of qualitative conclusions, whose interpretations and redefinitions result in the (re)conceptualization of the original conclusions^11^, thus meeting the objective of this article.

The guiding question for this review was: what are the evidences found by qualitative studies on the work of Brazilian CHA who are linked to the FHS, from 2004 to 2014? To this end, we searched BIREME in March 2015 using the descriptors “*Agente Comunitário de Saúde*” and “*Trabalho*”, in Portuguese, crossed from the Boolean operator AND, and we filtered Brazilian articles published between 2004 and 2014. Thus, we retrieved 129 articles, being 124 indexed in the Lilacs database and five in the Medline database.

This result was exported to the Mendeley reference manager, which detected six duplicate documents. Next, we started the screening process, which was carried out by the lead author with the supervision of the co-authors to minimize biases from the presence of only one evaluator. We screened the articles that presented original qualitative results and that addressed the work of CHA linked to the FHS.

The exclusion criteria considered the type of methodological approach used in studies, study subjects, and nature of the article. Thus, we removed quantitative or quanti-qualitative research articles, essays, debates, literature reviews, reports of experiences, as well as those that did not include CHA as research subjects.

Following these criteria, the articles were selected in three stages: analysis of the titles, analysis of abstracts, and analysis of full texts. Thus, we removed 46 articles analyzing the title, 34 analyzing the abstract, and we fully analyzed 44 of them. In this last phase, we also removed 11 articles. The Figure shows the flow of article selection, whose result was the corpus of this meta-synthesis.

**Figure:**
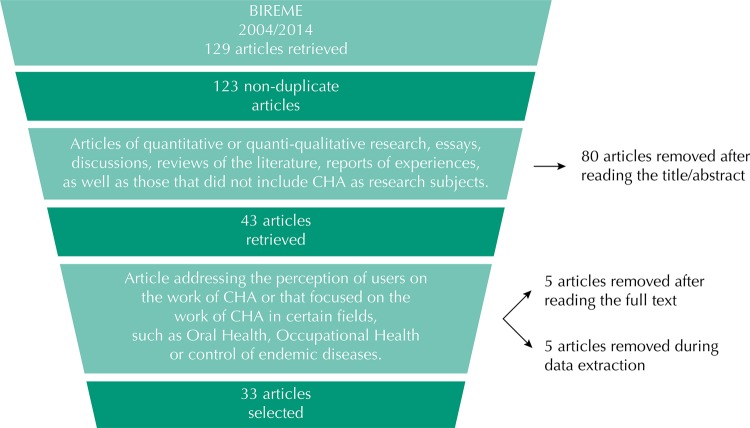
Flow of the selection process of the articles in the different phases of the meta-synthesis of the perception of CHA on their work.

BIREME: Latin-American and Caribbean Center on Health Sciences Information; CHA: Community Health Agent

## ANALYSIS OF RESULTS

The Box summarizes the 33 articles selected and their main results. We verified the following distribution of these studies among the Brazilian regions: twenty in the Southeast, seven in the Northeast, three in the South, two in the Midwest, and one that covered the North, Midwest, and Southeast at the same time. Regarding this aspect, we highlight a discrepancy in the number of studies carried out in the Southeast region compared to the other Brazilian regions, which is consistent with an article that has shown that health research in Brazil is concentrated in the Southeast region^14^.

**Box:** Summary of the information from selected studies for the meta-synthesis of the perception of Community Health Aagents about their work.

Authors/year	Region	Subjects of the research	Procedure for data collection	Subject
Baralhas M, Pereira MAO^1^ (2011)	Southeast	CHA	Interview	Representation of CHA on their care practices
Barbosa RHS, Menezes CAF, David HMSL, Bornstein VJ^2^ (2012)	Southeast	CHA	Focus group	Relationship between the gender dimension and the work of CHA
Binda JB, Bianco MF, Sousa EM^3^ (2013)	Southeast	CHA	Observation, discussion groups, and interview	Analysis of the work processes of CHA
Bornstein VJ, Stotz EM^4^ (2008)	Southeast	CHA, physicians, and nurses of FHS	Interviews, documentary analysis, and participant observation	Characterization of the types of mediation present in the work of CHA
Carli R, Costa MC, Silva EB, Resta DG, Colomé ICS^5^ (2014)	South	CHA	Interview	Perceptions of CHA on the reception and bonding practices
Coriolano MWL, Lima LS^6^ (2010)	Northeast	CHA	Focus group	Description of the work process of CHA
Costa MC, Silva EB, Jahn AC, Resta DG, Colom ICS, Carli R^7^ (2012)	South	CHA	Interview	Analysis of the work process of CHA
Ferreira VSC, Andrade CS, Franco TB, Merhy EE^9^ (2009)	Northeast	CHA	Interview and focus group	Production of care, work process, technologies, and restructuring of the productive process of CHA
Filgueiras AS, Silva ALA^10^ (2011)	Southeast	CHA	Interview	Discussion of the facilitating and limiting aspects of the activities assigned to CHA
Fonseca AF, Machado FRS, Bornstein VJ, Pinheiro R^12^ (2012)	North, Midwest, and Southeast	CHA, work supervisors of CHA, and service managers	Interview and focus group	Analysis of the processes of evaluation of the work of CHA
Galavote HS, Franco TB, Lima RCD, Belizário AM^13^ (2013)	Southeast	CHA	Interview and observation	Work process of CHA
Gomes AL, Lima Neto PJ, Silva VLA, Silva EF^15^ (2011)	Northeast	CHA	Interview and observation	Relationship between the organization and the work process and mental health of CHA
Jardim TA, Lancman S^17^ (2009)	Southeast	CHA	Group of Work Psychodynamics	Discussion about living and working in the same community
Lara MO, Brito MJM, Rezende LC^18^ (2012)	Southeast	CHA, nurse, physician, nursing assistant, and users	Interviews	Analysis of the influence of the cultural practices present in the work of CHA
Lima AP, Corrêa ACP, Oliveira QC^19^ (2012)	Midwest	CHA	Interview and participant observation	Identification of the knowledge of CHA about PCIS instruments/records
Lopes DMQ, Beck CLC, Prestes FC, Weiller TH, Colomé JS, Silva GM^20^ (2012)	South	CHA	Focus group	Suffering and pleasure in the work of CHA
Martines WRV, Chaves EC^21^ (2007)	Southeast	CHA	Interview and observation	Representations of CHA about the vulnerabilities for suffering at work and the manifestations of this suffering in the performance of work
Nascimento GM, David HMSL^22^ (2008)	Southeast	CHA	Participant observation	Development of an instrument for risk assessment of the work of CHA
Oliveira AR, Chaves AEP, Nogueira JA, Sá LD, Collet N^23^ (2010)	Northeast	CHA	Questionnaire	Research on the satisfaction and limitation in the daily work of CHA
Oliveira DT, Ferreira PJO, Mendonça LBA, Oliveira HS^24^ (2012)	Northeast	CHA	Interview	Perception of CHA about their work process
Peres CRFB, Caldas Júnior AL, Silva RF, Marin MJS^25^ (2011)	Southeast	CHA	Interview	Analysis of the difficulties and facilities of CHA regarding teamwork.
Pinheiro RL, Guanaes-Lorenzi C^26^ (2014)	Southeast	CHA	Groups of discussion	Perception of CHA about their practices with social networks
Queirós AAL, Lima LP^28^ (2012)	Northeast	CHA, managers, legislators, social movement, movement of CHA and popular movement, researcher	Interview	Analysis of the social practice of the work of CHA
Rosa AJ, Bonfanti AL, Carvalho CS^30^ (2012)	Midwest	CHA and users	Groups of work psychodynamics, interview, and observation	Analysis of the relationship between work and psychological distress of CHA
Sakata KN, Mishima SM^31^ (2012)	Southeast	CHA, dental office assistant, nursing assistant, dental surgeon, nurse, physician, and health unit manager	Interview and participant observation	Analysis of social relationships between CHA and family health teams
Santos LFB, David HMSL^32^ (2011)	Southeast	CHA	Interview	Identification of the occupational stress factors reported by CHA and analysis of their relation with possible health effects
Schmidt MLS, Neves TFS^33^ (2010)	Southeast	CHA	Focus groups	Analysis of the aspects of the implementation of the Family Health Program (FHP) as a primary public policy in the struggle with the hegemonic medical-care model in the country
Sossai LCF, Pinto IC, Mello DF^36^ (2010)	Southeast	CHA and users	Interviews	Presentation of the work of CHA from the perspective of users and CHA
Souza LJR, Freitas MC^37^ (2011)	Northeast	CHA	Interview and participant observation	Analysis of workplace violence in CHA
Trapé CA, Soares CB^39^ (2007)	Southeast	CHA	Focus groups and interviews	Conceptions of health education present in the work of CHA
Vilela RAG, Silva RC, Jackson Filho JM^40^ (2010)	Southeast	CHA and managers	Interview and observation	Analysis of the relationship between complaints of suffering and the conditions of work of CHA and proposed measures to modify them
Wai MFP, Carvalho AMP^41^ (2009)	Southeast	CHA	Interview	Perception of CHA on events that cause overload and coping strategies used by them.
Zanchetta MS, Leite LS, Perreault M, Lefebvre H^42^ (2005)	Southeast	CHA	Individual and group interviews. Observation	Perception of CHA on barriers in the work practice

CHA: Community Health Agents; PCIS: Primary Care Information System; FHS: Family Health Strategy

The analysis of the results of the articles produced three thematic categories, presented below, that became the axis to understand the problem explored in this meta-synthesis.

### Characteristics of the Work of CHA

The work of CHA is more permeated by the political and social dimensions of health work^6^ ^,^ ^31^, with predominant use of light technologies, such as communication^6^ ^,^ ^7^, reception and bond^1^ ^,^ ^5^ ^,^ ^7^ ^,^ ^9^ ^,^ ^17^, dialog^4^, and listening^1^ ^,^ ^4^ ^,^ ^26^.

Some studies^4^ ^,^ ^9^ point out that the main input of the work of CHA is the knowledge obtained in the contact with families and, as far as this contact is concerned, research studies state that home visits are a privileged stage for its development^1^ ^,^ ^4^ ^,^ ^5^ ^,^ ^10^.

Vilela et al.^40^ state that home visits are primary activities to build the relationship between CHA and users, and they also represent the main means to promote the health of the surrounding community. From the managerial point of view, home visits are valued operations that count as production of the unit.

One study^12^ has specifically addressed the evaluation of the work of CHA, evidencing that this evaluation is based on a quantitative bias based on the achievement of goals that predominantly encompass tasks related to the biomedical field.

### Problems Related to the Work of CHA

The main challenges related to the work of CHA evidenced in the studies are described below.

#### Lack of limits in the work of CHA

Thirteen articles have addressed the lack of limits of the actions of CHA summarized in two issues: idealization of the work and lack of limits of the attributions of this professional.

Regarding the idealization of the work, studies have shown that CHA perceive as their mission the solution of all the issues of the families and the community they serve, idealizing their practices and disregarding the importance of other resources to carry out their actions^1^ ^,^ ^21^. Studies show that CHA understand their work as a vocation based on values of friendship, solidarity, voluntariness, and charity, which are based on the perception about their attributions as elements that contribute to the difficulty in delimiting their role^1^ ^,^ ^2^ ^,^ ^21^ ^,^ ^36^ ^,^ ^42^.

The lack of limits of the work of CHA can be understood both in studies that highlight the lack of clarity of their duties^20^ ^,^ ^21^ and in studies that emphasize the excess of planned functions^6^ ^,^ ^15^ ^,^ ^20^ ^,^ ^22^ ^,^ ^23^ ^,^ ^30^. For the excess of functions, the CHA perform tasks that do not concern them, such as: work at the reception^28^ ^,^ ^36^ ^,^ ^40^, scheduling of appointments^1^ ^,^ ^15^ ^,^ ^28^ ^,^ ^36^, organization of folders and medical records^36^, control of materials and warehouse and cleaning service^36^, delivery of referrals to specialists^15^ ^,^ ^31^, sending of messages from the health service to users^31^, and feeding of children in the absence of parents^42^.

In addition to the tasks that are not the responsibility of CHA, other functions are gradually incorporated into their role, officially, such as the weighing of families for their re-registration in the government program *Bolsa Família* and actions to prevent and combat dengue fever^15^.

This framework reinforces that CHA are seen as a multipurpose workers, given the lack of definition of the limits of their professional duties and the idealization of the role, and thus their scope of action is constantly extended.

#### Precarious working conditions

Another problem related to the activities of CHA is their precarious working conditions, identified in thirteen articles: explicitness of the fragility of the employment relationship^1^ ^,^ ^3^ ^,^ ^33^, exposure to work hours that go beyond the opening hours of the health unit and which invades the private life^1^ ^,^ ^6^ ^,^ ^7^ ^,^ ^17^ ^,^ ^40^ ^,^ ^41^, care for a greater number of families than what is advocated^35^, exposure to unhealthy working conditions^30^ ^,^ ^41^, low pay and absence of social protection^1^ ^,^ ^4^ ^,^ ^7^ ^,^ ^30^, poor recognition of work by managers, peers, and users^2^ ^,^ ^12^ ^,^ ^20^ ^,^ ^24^ ^,^ ^26^ ^,^ ^32^, and precariousness of the system^17^ ^,^ ^40^.

As a consequence of this precariousness, two studies have shown that CHA view the profession as a temporary activity, since there is no prospect of transformation of the work situations or a planned career plan^20^ ^,^ ^30^.

The precariousness of the work of CHA is also related to the fragility of the health system, which cannot adequately meet the demand of users, with a lack of opening for appointments and examinations, materials, and medications. This situation affects the relationship between CHA and the community they serve, since many users attribute to them the administration of the lack of resources of the health unit and the system, considering their responsibility for the front line in the care of the population^17^ ^,^ ^40^.

#### Relationship with the community

As for the relationship of CHA with the community, the most reported problems were: obligation to live in the community where they work, coexistence with community problems, exposure to violence, and relationship with users.

The CHA need to live in the community they serve, which is a problem as these workers have their private life exposed when sought outside working hours, on weekends, and in living spaces in the neighborhood, such as church and fairs, thus overloading them^6^ ^,^ ^10^ ^,^ ^17^ ^,^ ^20^ ^,^ ^41^. In this way, these professionals have an uninterrupted involvement with the users of the health service. Jardim and Lancman^17^ emphasize that, just as CHA enter the private world of users, the private world of these workers is also invaded by the community and its problems, as they are literally unable to keep a distance from the population for which they are responsible.

The second subject of this category addresses studies that point to the fact that CHA generally work with poor populations living in peripheral regions. In these regions, social problems are more acute, increasing the emotional load related to work, both because of the difficulty in managing these issues and because CHA often share the same problems as they also live in these communities^4^ ^,^ ^6^ ^,^ ^17^ ^,^ ^20^ ^,^ ^23^ ^,^ ^41^.

The third subject related to the relationship between CHA and the community refers to the exposure of these workers to violence. Studies carried out in large urban centers have shown that these professionals work in regions where violence is pronounced, especially that related to organized crime and drug trafficking^32^ ^,^ ^37^ ^,^ ^42^.

Therefore, because they work and live in the same territory, CHA are more vulnerable to situations of conflict compared to the other FHS workers who do not circulate as much. In this sense, studies^6^ ^,^ ^37^ state that CHA generally need to build strategies to cope with these situations, corroborating the findings of Jardim and Lancman^17^, who report that CHA avoid reporting to the police or Child Protective Council for fear for their safety.

In line with this issue, the problem of the relationship between CHA and users is manifested. According to Bornstein and Stotz^4^, CHA perceive that users expect a more viable access to health services, which is similar to the result of other studies^10^ ^,^ ^17^. However, when these professionals cannot respond to the demands of users for appointments, medications, exams, or access to other services, users lose their trust on CHA without considering that the lack of access is a matter of how the system works^1–5,7,22,23,30,32,33,40,41^. Jardim and Lancman^17^ have found that the credibility of CHA in the community is directly associated to the resolution of the demands of users and this credibility can be hampered by aspects related to the structuring of the service and the inoperability of the health system.

In this sense, another dimension that is related to this issue has been identified by Santos and David^32^ and refers to the violence present in the work of CHA, which emerges from the relationship of these professionals with the community. These authors have observed that the frustration of users to demands not met can also turn into verbal aggression or intense psychological pressure.

#### Fragility in the professional qualification of CHA

The CHA consider their professional training insufficient and the main perceived gaps were: excess of standardization of contents that address predominantly technical-scientific subjects and that do not include data on the local reality, lack of focus on theoretical and practical aspects that could assist them in coping with issues of the daily work, such as the management of family and social problems, and, finally, restriction of the work hours offered for such activity^1^ ^,^ ^4^ ^,^ ^7^ ^,^ ^10^ ^,^ ^19^ ^,^ ^36^ ^,^ ^39^.

#### Bureaucratization of the work

The bureaucratization of the work of CHA is another evidence highlighted in the studies included in this meta-synthesis, and it is related, in the perception of these workers, to the collection of data on the surrounding population, especially those related to diet and use of the Primary Care Information System (PCIS)^19^.

In this sense, authors^19^ state that CHA have difficulties in identifying, naming, and describing the records they need to fill in to feed the PCIS, and they also do not understand the variables, terms, and pathologies that make up these instruments. As a consequence, tasks related to health surveillance, notwithstanding their importance to the FHS, focus on the work of CHA as mere statistical data collection activities, with little meaning for these workers^1^ ^,^ ^32^ ^,^ ^36^ ^,^ ^40^.

Other aspects regarding the perception of bureaucratization were listed in the study of Nascimento and David^22^, which indicate the presence of the following characteristics in the work process of CHA: standardization, great number of prescriptions, and organization according to the logic of process divisions and hierarchy.

In this perspective, bureaucratization starts to permeate even the tasks that are not administrative, given the predominance of the logic to count procedures, instead of assessing the quality of the care provided^9^ ^,^ ^12^ ^,^ ^19^.

#### Problems in the relationship with the team

Teamwork was identified as a limiting factor in the work of CHA, when there is a lack of articulation with other professionals and an inflexibility of relationships produced by the work organization that hinders the exchange between players outside the spaces established for this end^7^ ^,^ ^13^ ^,^ ^41^ ^,^ ^42^.

Peres et al.^25^, when analyzing the perceptions of CHA on teamwork, indicate that these workers feel as the weakest link in the relationship with other FHS professionals. Such situation is in line with the findings of other authors^3^ ^,^ ^9^ ^,^ ^40^ who have identified a hierarchy of knowledge in the FHS that gives to CHA an unequal role in the planning and decision making of interventions.

When analyzing the work of CHA in the interface with the teams, a dysfunction was found in the relation between CHA and teams, from the different ways of approaching the problems of users. In this sense, it is common for teams not to value what CHA raise as a priority in meeting the needs of users^40^.

In addition, the organization of work in the FHS is guided by the achievement of goals, excess of activities, and little availability of time for the exchange between professionals, which hinders the establishment of teamwork and is negatively reflected on the work of CHA^31^ ^,^ ^41^ ^,^ ^42^.

Other perceived difficulties are caused by “personal differences; difficulty in visualizing all actions; lack of flexibility, communication, cooperation, responsibility, and horizontality of actions” (Peres et al.^25^, p.908).

## Facilitating Aspects of the Work of CHA

We describe below the positive aspects that act as facilitators of the work of CHA identified by the selected studies.

### Recognition of the work by families/community and resolubility

The most recurrent positive aspect in this meta-synthesis was the recognition of the families in relation to the work of CHA. Thus, several authors^1^ ^,^ ^6^ ^,^ ^7^ ^,^ ^13^ ^,^ ^23^ ^,^ ^36^ ^,^ ^42^ have pointed out that CHA feel satisfied with their work when they see that they are useful to the community, when there has been a change in the health conditions of users, or when families recognize their competence and commitment. Two studies^2^ ^,^ ^20^ reinforce these findings, adding that the recognition of the assisted population is a motivating factor for CHA, strengthening their self-esteem and contributing to the conformation of their identity.

The resolubility of CHA has been identified in the study by Lopes et al.^20^ as “possibility to solve the problems of users and to verify that the work carried out is improving the health conditions of the community” (p.635). In this context, resolubility is identified as a positive aspect related to the work of CHA, since this characteristic is linked to the feeling of gratification and utility, thus contributing to the professional satisfaction of these workers^23^ ^,^ ^36^.

### Bonding with families and community

Seven studies included in this meta-synthesis show that CHA perceive the bond with the user as a necessary condition for their work to happen, because, according to some authors^1^ ^,^ ^5^ ^,^ ^13^ ^,^ ^31^ ^,^ ^42^, this bond is intimately related to the mission of being the link between health professionals and the community. The construction of the trust and credibility of CHA among users has been identified in five studies^1^ ^,^ ^5^ ^,^ ^17^ ^,^ ^18^ ^,^ ^31^, as dimensions that favor the establishment of the bond. This is because they allow the worker to approach problems that extrapolate the biological dimension, despite being part of the health-disease process, such as situations of family or community conflict, domestic violence, poverty, sexual abuse, child neglect, ill-treatment of older adults, trafficking, and use of drugs. Such situations are complex and they cannot always be accessed without building a trust relationship between users and CHA.

### Working together with peers

Peer-to-peer activities were identified as facilitators when they were established by the positive relationships between CHA and other staff members, allowing the horizontal discussion of daily problems and allowing the sharing of work strategies^9^ ^,^ ^25^ ^,^ ^31^. Such a construction is favored in team meetings from the perspective of CHA^24^.

In fact, according to Filgueiras and Silva^10^, the positive aspects of teamwork are the support for the actions of CHA, as the integration with the other professionals allows the collective construction of coping mechanisms for the problems identified.

### Formal work close to the residence

The last positive trait in the work of CHA identified in the studies analyzed concerns the formal employment relationship. This situation has been evidenced in the study by Sossai et al.^36^, which show the rare presence of job vacancies that comply with the norms of the Labor Law Consolidation in the region of the study. This distinguishes the work of CHA from the other workers, as CHA can combine formal employment with living close to their residence, meeting the findings of Zanchettaet al.^42^

In addition, two other studies^2^ ^,^ ^3^ have shown that work close to home is perceived as advantageous, especially for women, who are most of the CHA. This situation enables the combination of the care of the family with the professional work.

## DISCUSSION

We aimed to collect and analyze evidence on the work process of CHA with this meta-synthesis. We analyzed 33 articles, which allow us to perceive the characteristics of the profession, as well as problems and positive aspects related to professional work. In this sense, we emphasize that the academic production that addresses the subject of the work of CHA is useful in pointing this evidence.

The duties of CHA need to be reviewed in order to better define their role and size their actions according to the resources available, avoiding, above all, the abuse of functions.

We need to overcome the precariousness of the work of CHA by building a career plan that values not only the knowledge accumulated over time but also the strategic role they play in the consolidation of the FHS.

Another important issue raised in this meta-synthesis refers to the need to rethink the organization of work within the FHS, incorporating demands related to the specificities of the work of CHA. In this sense, the organization of the work of FHS teams must be reviewed to allow CHA to have a space of dialog strengthened with the other members of the team. In fact, horizontal teamwork, with integration among members, is positively reflected in the work of CHA.

Regarding the organization of the work, we can observe the need to rethink the bureaucratic tasks inherent to CHA. This would be done, on the one hand, by giving a meaning to the filling of the reports used to collect population data, because as Lima et al.^19^ have observed, the CHA have difficulty recognizing the usefulness of these instruments. On the other hand, we can mention that although the work in the FHS and, in particular the work of CHA, is based on an expanded concept of health, in the principles of the PHC which is parametrizated qualitatively, we can observe the prevalence of the normative logic that organizes the work hierarchically and using processes.

This fact is also reflected in how the work done is evaluated, which is given by the counting of procedures and not by the quality of the care. Given this situation, we need to urgently rethink models that evaluated the work of CHA that are in line with the tools and values recommended for the FHS while also contemplating the specificities of the profession.

Regarding the issue of living with the community problems, the fact that CHA live in the same territory where they work is a challenge. Although this exposes workers to violence and to the invasion of their private life, the most significant set of knowledge of the CHA is structured in the contact with the community. In addition, we observed that the work close to the residence also benefits these workers.

Regarding the fragility in the training of the CHA, Ordinance 253, of September 25, 2015, recently established the Introductory Course for CHA, which standardized the minimum work hours for this training and defined the basic curricular components.

In conclusion, regarding the distribution of research in Brazil, we verified a predominance of studies carried out in the Southeast region and in large urban centers. In this sense, new studies can be carried out in Brazilian regions with different realities, such as those that attend to riverine or rural populations, where the work of CHA assumes other characteristics. Regarding the meta-synthesis carried out, we presented an overview of the difficulties and positive aspects that are present in the daily work of CHA. Given this, this review posed two challenges for future research. The first one refers to how public policy makers need to appropriate the research results and the second one refers to the need to invest in studies that are designed to generate solutions for the difficulties faced by CHA in their work.
